# Characterization of a Novel Acid-Stable Chitosanase from *Lentinula edodes* Suitable for Chitooligosaccharide Preparation

**DOI:** 10.3390/foods13193127

**Published:** 2024-09-30

**Authors:** Yanxin Wang, Yujie Zhao, Jingchen Li, Haobo Zong, Ying Chen, Jinyu Zhou, Xinlian Li, Xianfeng Ye

**Affiliations:** 1 College of Agriculture and Biology, Liaocheng University, Liaocheng 252000, China; wangyanxin@lcu.edu.cn (Y.W.);; 2 Key Laboratory of Agricultural Environmental Microbiology, Ministry of Agriculture and Rural Affairs, College of Life Sciences, Nanjing Agricultural University, Nanjing 210095, China

**Keywords:** chitosanase, *Lentinula edodes*, acid-stable, chitosan, chitooligosaccharides, characterization

## Abstract

As high-value chitosan derivatives, chitooligosaccharides (COSs) with biodegradable, biocompatible, nontoxic, antimicrobial, and antioxidant activities have been widely applied in food-related fields. Chitosanases can hydrolyze chitosan to produce COSs. Herein, a chitosanase (*Le*Cho1) from *Lentinula edodes* was successfully expressed in *Escherichia coli* and was then purified and characterized. *Le*Cho1 had a low sequence identity with other chitosanases reported from the GH75 family. The recombinant protein showed a molecular mass of 27 kDa on SDS-PAGE. *Le*Cho1 preferentially hydrolyzed chitosan with a high degree of deacetylation (DDA) and exhibited maximal activity (71.88 U/mg) towards 95% DDA chitosan at pH 3.0 and 50 °C. It possessed good stability at pH 2.0–6.0 and temperatures below 45 °C. Its hydrolytic activity was remarkably enhanced by the metal ion Mn^2+^ at 1 mM, while it was totally inhibited by 1 mM Fe^3+^ or 10 mM EDTA. Its *K*_m_ and *V*_max_ values were 0.04 μM and 76.81 μmol·min^−1^·mg^−1^, respectively, indicating good substrate affinity. *Le*Cho1 degraded chitosan into COSs with degrees of polymerization (DPs) of 2–5, while it had no action on COSs with DPs of less than 5, revealing its endo-chitosanase activity. This study proved that chitosanase *Le*Cho1 is a promising candidate in the industrial preparation of COSs due to its excellent properties.

## 1. Introduction

Chitosan, as the *N*-deacetylated product of chitin, is a linear heteropolysaccharide made up of two monosaccharides, D-glucosamine (GlcN) and N-acetyl-D-glucosamine (GlcNAc), connected through β-1,4-glycosidic bonds [1]. Chitosan has many biological activities, such as non-toxicity, biocompatibility, biodegradability, antimicrobial, antitumor, high blood pressure inhibition, and an immune-enhancing effect [2]; however, its applications have been limited due to its poor solubility at neutral pH and high viscosity. At present, chitosan oligosaccharides (COSs) with degrees of polymerization (DPs) in the range of 2–20 [3], obtained by the depolymerization of chitosan, have received extensive consideration in many sectors due to their exceptional physicochemical properties, including high water solubility and low viscosity [4]. Especially in food-related industries, chitosan and its derivative COSs have been implemented to improve the quality and shelf-life of various food and food products. For example, they have served as potential natural antioxidants to inhibit lipid oxidation in foods rich in polyunsaturated fatty acid and relieve the off-flavor and off-odor of fatty foods [5], and COSs have exhibited higher activity in lowering lipid oxidation due to the higher proportion of –OH groups and NH_2_ groups after the hydrolysis of chitosan [6]. Furthermore, they were also used as potential antimicrobial agents to suppress microbial spoilage and have been applied in beer brewing and food processing [7,8]; chitosan with lower molecular weight and COSs have been reported to have higher antimicrobial activity due to their unobstructed contact with bacterial cells [9]. Additionally, they possess excellent film-forming properties and have been employed as potential packing materials to provide barriers to aroma, oxygen, oil, and moisture and to enhance the appearance and flavor properties of preserved foods [10].

In general, COSs are prepared by three methods, including the chemical method, which involves acid hydrolysis and oxidative degradation [11] and which results in difficulty in separating and purifying the products, resulting in environmental pollution and the production of harmful by-products. The physical method involves microwave degradation, ultrasonic treatment, and ultraviolet irradiation [12], which require high energy consumption and have a low product yield. The enzymatic degradation method utilizes cellulases or chitosanases [13] and has some difficulties in industrialization due to its high cost in terms of production and harsh conditions in reaction. Compared to the first two methods above, enzymatic conversion to produce COSs has recently drawn widespread attention because of its environmental friendliness, the controllability of the final product, and the efficiency of production. Many enzymes reported are able to hydrolyze chitosan into COSs, such as cellulases, proteases, lipases, chitinases, and chitosanases [14]. Among them, chitosanases are a specific enzyme involved in the hydrolysis of chitosan; thus, exploring a new chitosanase with high activity and high stability under a mild reaction environment is of great significance in the preparation of COSs.

As glycoside hydrolases (GHs), chitosanases can specifically degrade β-1,4-glucoside bonds located in chitosan and produce COSs or glucosamine (GlcN) [15]. Chitosanases have been found in many organisms, such as bacteria, fungi, cyanobacteria, and plants [16]. The enzymes have been classified into three subclasses on the basis of the cleavage positions’ specificity in the substrate chitosan, such as subclass I cleaving GlcN–GlcN and GlcNAc–GlcN linkages, subclass II cutting GlcN–GlcN linkages or GlcN–GlcN linkages, and subclass III cracking GlcN–GlcNAc linkages [17]. Furthermore, these enzymes have also been divided into endo-chitosanases, which could hydrolyze the β-1,4-glycosidic bonds of chitosan and produce a mixture of COSs with various DP values, and exo-chitosanases, which are capable of cutting GlcN residues individually from the chitosan’s non-reducing termini to release a series of monosaccharides. Most of the reported chitosanases belong to the endo-enzymes group. Additionally, based on the similarity of amino acid sequences, chitosanases have been categorized into six glycoside hydrolase (GH) families in the Carbohydrate-Active Enzymes database (CAZy, www.cazy.org, updated in September 2024), including the GH5, GH7, GH8, GH46, GH75, and GH80 families [18], among which, the GH46, GH75 and GH80 families are all made up of chitosanases. Chitosanases from fungi have been reported to be mainly distributed in the GH75 family, while those from bacteria have been found in the GH46 family [19]. In addition, chitosanases with different biological functions have been found in different organisms. For example, chitosan-degrading microorganisms utilize chitosanases to acquire extracellular nutrition [20], fungi from Zygomycetes secrete chitosanases to hydrolyze the structural components of cell walls for morphogenesis [21] and plants employ chitosanases as a defensive weapon against phytopathogens [22]. In addition to this, chitosanases have potential applications in many areas, such as the bioconversion of marine crustacean biomaterials [23], the preparation of fungal protoplasts [21], the biocontrol of plant pathogens [24] and the manufacture of COSs [25]. Presently, various chitosanases from different sources have been reported; however, only a few have been used in industrial production. Therefore, screening for a new chitosanase with excellent properties is still urgently needed.

Previous studies reported that a putative chitosanase from *Lentinula edodes* (*Le*Cho1) exhibited up-regulated transcription levels post-harvest, and it was proposed that this cell wall degradation-related enzyme was closely related to fruiting body autolysis [26,27]. However, there was no detailed biochemical characterization of this protein. In this study, *Le*Cho1 was heterologously expressed in *Escherichia coli* with a 6×His tag and purified using Ni-NTA affinity chromatography. The purified protein was then subjected to detailed biochemical characterization, and its hydrolytic pattern was determined. The results of this study illustrate the outstanding characteristics of *Le*Cho1 in the preparation of chitoologosaccharides (COS), laying a crucial foundation for its industrial application.

## 2. Materials and Methods

### 2.1. Polysaccharides

Chitosan oligosaccharides (≥95% purity), including chitobiose hydrochloride (ZB-10006), chitotriose hydrochloride (ZB-10007), chitotetraose hydrochloride (ZB-10008), chitopentaose hydrochloride (ZB-10009), and chitohexaose hydrochloride (ZB-10010), were obtained from Zhenzhun Biotechnology Co., Ltd. (Shanghai, China). Chitosan with different degrees of deacetylation (DDA; 70%, 80%, and 95%), chitin, microcrystalline cellulose, and sodium carboxymethylcellulose were procured from Maclin Biochemical Technology Co., Ltd. (Shanghai, China).

### 2.2. Strains and Plasmids

The genomic DNA of *L. edodes* cultivation strain H600 was stored in our laboratory and was used as a template to clone the chitosanase gene. *E. coli* BL21 (DE3) was used to express the recombinant protein. The vector pET29A was employed to construct the recombinant expression plasmid.

### 2.3. Sequence Analysis

The amino acid sequences of chitosanase *Le*Cho1 were derived from the genome of *L. edodes* in GenBank (Accession ABH80469.1). Potential signal peptide sequences were predicted using the SignalP-5.0 server (https://services.healthtech.dtu.dk/services/SignalP-5.0/, accessed on 18 February 2022). The theoretical molecular weight and isoelectric point (pI) of *Le*Cho1 were obtained through the Expasy server (https://web.expasy.org/protparam/, accessed on 15 May 2022). Based on amino acid sequences of *Le*Cho1 and previously reported chitosanases from the GH46, GH75, and GH80 families, a phylogenetic tree was constructed in MEGA v.6.06 software with 1000 bootstrap iterations using the neighbor-joining method. All the protein sequences were obtained from NCBI. The sequence identity between *Le*Cho1 and other chitosanases from the GH75 family was determined by amino acid sequence alignment in Clustal W version 2.0. The alignment result was processed using ESPript 3.0 (http://espript.ibcp.fr/ESPript/cgi-bin/ESPript.cgi, accessed on 26 September 2022).

### 2.4. RNA Extraction and cDNA Synthesis

The mycelia of *L. edodes,* preserved in our labs, were inoculated onto the potato dextrose agar (PDA) medium and placed in an incubator at 26 °C for 12 d in the dark. The resulting fresh mycelia were collected to extract total RNA using a Spin Column Fungal Total RNA Purification Kit (Sangon, Shanghai, China) according to the manufacturer’s recommendation. The synthesis of cDNA was carried out using a HiScript II Q Select RT SuperMix for qPCR (+gDNA wiper) Kit (Vazyme Biotech Co., Ltd., Nanjing, China) following the technical manual. The resulting cDNA was stored at −80 °C until further use. 

### 2.5. Cloning, Expression and Purification of LeCho1

The nucleotide sequences encoding *Le*Cho1 without the signal peptide were amplified using the forward primer (TAAGAAGGAGATATACATATGGCGCCCATTCCTAGAAATCTTCGGA) and the reverse primer (TAAGAAGGAGATATACATATGGCGCCCATTCCTAGAAATCTTCGG; the cleavage sites are underlined). The product was ligated with the plasmid pET29A digested with *Nde* I and *Xho* I using the ClonExpress II/One Step Cloning Kit (Vazyme, Nanjing, China), and transferred into competent cells of *E. coli* BL21 (DE3). Transformants were screened by colony PCR and confirmed using Sanger sequencing. For protein expression, the cells were grown in LB medium containing kanamycin (50 mg/L) at 37 °C and 220 rpm. When the optical density at 600 nm (OD_600_) of the culture reached 0.5–0.6, isopropyl-β-D-thiogalactopyranoside (IPTG) with a final concentration of 1 mM was added to induce expression of the target protein. Then, the induced cells were further incubated at 16 °C for 20–24 h, after which they were collected by centrifugation at 12,000× *g* for 5 min. Following sonication at 4 °C, the recombinant *Le*Cho1 containing a 6×His tag was loaded onto Ni^2+^-nitrilotriacetic acid (NTA) resin, and the purified fractions were collected and concentrated by ultrafiltration. The purity of the harvested protein was detected using 12% acrylamide sodium dodecyl sulfate-polyacrylamide gel electrophoresis (SDS-PAGE), and the bands were stained with Coomassie G-250. The concentration of the purified protein was determined using the Bradford method with bovine serum albumin (BSA) as the standard [28].

### 2.6. Hydrolytic Activity Assay 

The hydrolytic activity of *Le*Cho1 was measured using the 3,5-dinitrosalicylic acid (DNS) method [29]. The amount of reducing sugar released from the substrates (chitosan, chitin, microcrystalline cellulose, and sodium carboxymethylcellulose) was determined by detecting the absorbance at 540 nm (OD_540_) of the supernatant from the reaction mixture using GlcN as the standard. The reaction mixture (200 μL), including 2 μg *Le*Cho1 and 0.5% (*w*/*v*) substrate in 50 mM sodium acetate buffer (NaAc-HAc, pH 5.0), was incubated at 40 °C for 30 min, after which DNS reagent (200 μL) was added and placed at 100 °C for 10 min. After cooling to room temperature, the supernatant of the above mixture was collected by centrifugation, and its absorbance at OD_540_ was measured. All reactions were conducted in triplicate. One unit (U) of chitosanase activity was defined as the amount of enzyme liberating 1 μmol of reducing sugar per minute under the above conditions with glucosamine as standard.

### 2.7. Biochemical Characterization of Recombinant LeCho1

Chitosan (0.5% *w*/*v*, 95% DDA) was used as the substrate to examine the enzymatic properties of *Le*Cho1. The optimal pH of *Le*Cho1 was determined by measuring the hydrolytic activity of the enzyme in 50 mM buffers at pH values in the range of 2.0–9.0, including Gly-HCl buffer (pH 2.0–3.0), NaAc-HAc buffer (pH 3.0–6.0), sodium-phosphate buffer (PBS, pH 6.0–7.0), and Tris-HCl buffer (pH 7.0–9.0). To assay the pH stability, purified *Le*Cho1 (2 μg) was incubated in different buffers at 4 °C for 12 h, after which the residual chitosanase activity was detected using chitosan (0.5% *w*/*v*, 95% DDA) dissolved in 50 mM Gly-HCl buffer (pH 3.0) at 40 °C for 30 min.

The optimal temperature of *Le*Cho1 was evaluated by examining the hydrolytic activity of the enzyme at various temperatures (20–80 °C) for 30 min in 50 mM Gly-HCl buffer (pH 3.0). To determine the thermal stability, purified *Le*Cho1 in Gly-HCl buffer (pH 3.0) was placed at 20, 30, 40, 50, 60, 70, and 80 °C for 1 h and cooled to room temperature, after which the residual activity was determined at 40 °C and was detected under the standard condition.

The influence of metal ions on chitosanase activity was assessed by incubating purified *Le*Cho1 with EDTA (1 mM and 10 mM) or 1 mM individual metal ions (Na^+^, K^+^, Ba^2+^, Cu^2+^, Ca^2+^, Co^2+^, Fe^2+^, Mn^2+^, Mg^2+^, Ni^2+^, Zn^2+^, Fe^3+^, Al^3+,^ and Cr^3+^) in Gly-HCl buffer (50 mM, pH 3.0) containing chitosan (0.5% *w*/*v*, 95% DDA) at 40 °C for 30 min. The hydrolytic activity of *Le*Cho1 without any additives was defined as 100%.

The kinetic parameters of the protein were determined by measuring the initial reaction velocity at various concentrations of 95% DDA chitosan (0.1%, 0.2%, 0.4%, 0.5%, 0.6%, and 0.8%, *w*/*v*). Reactions were performed in Gly-HCl buffer (50 mM, pH 3.0) at 40 °C for 40 min. The Michaelis–Menten constant (*K*_m_) and the maximal catalytic rate (V_max_) were determined using Lineweaver–Burk plots.

### 2.8. Analysis of Hydrolysis Products

The hydrolytic pattern of *Le*Cho1 was determined by analyzing the hydrolysates of substrates, including COSs [(GlN)_2_ to (GlN)_6_] and chitosan (0.5% *w*/*v*, 95% DDA). Reaction mixtures (200 μL), including 2 μg *Le*Cho1 and 0.5% (*w*/*v*) chitosan or 0.1 μM COSs in 50 mM Gly-HCl buffer (pH 3.0), were incubated at 40 °C for different time periods (0, 0.5, 1, 2, 4, 6, and 12 h). After the indicated time, the mixtures were immediately placed at 100 °C for 10 min to stop the reaction, then centrifuged at 12,000× *g* for 5 min to remove the denatured protein.

The distribution of hydrolysate components was analyzed using thin-layer chromatography (TLC) according to the method described by Wang et al. (2021) [15]. Briefly, the above supernatants were spotted onto the TLC plate (Silica gel 60 F254 aluminum sheet, Merck, Darmstadt, Germany) and developed in a solvent comprising ammonium hydroxide/water/isopropanol (0.3:2.7:7, *v*/*v*/*v*). The dried plate was sprayed with 0.5% ninhydrin reagent (dissolved in ethanol) and heated at 95 °C for 10 min to visualize the products.

In addition, hydrolysates of chitosan after 12 h of enzymolysis were analyzed using matrix-assisted laser desorption ionization-time-of-flight mass spectrometry (MALDI-TOF MS) [30] on a Bruker Autoflex time-of-flight mass spectrometer (Bruker Daltonics, Karlsruhe, Germany). During detection, 2,5-dihydroxybenzamide (DHB) (20 mg/mL DHB dissolved in 30% acetonitrile with 0.1% trifluoroacetic acid) served as the matrix to assist the ionization of hydrolysates. The acceleration voltage was set to 29 kV in reflection mode. All spectra were obtained in positive ion mode. The target mass range was an *m*/*z* ratio of 100/2000.

### 2.9. Statistical Analysis

All experiments were performed independently in triplicate. The values are shown as the means ± standard deviations (SD). To evaluate the variance and significant differences between the different treatments, a single-factor analysis of variance (one-way ANOVA) and Duncan’s test with a confidence level of *p* < 0.05 were performed using SPSS statistics software version 22.0 (IBM Corporation, Armonk, NY, USA).

## 3. Results

### 3.1. Sequence Analysis of LeCho1

**Figure 1:**
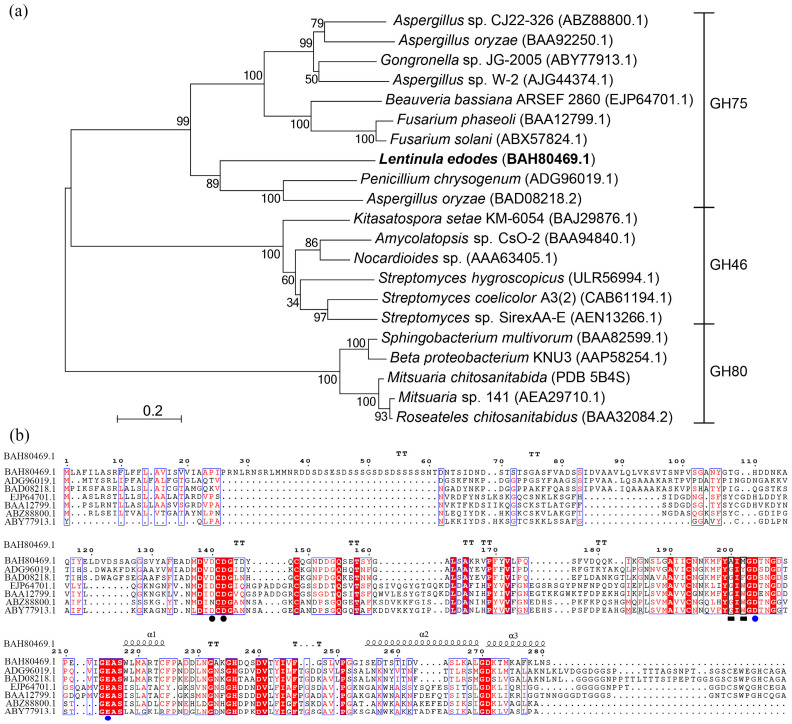
Sequence analysis of the chitosanase *Le*Cho1. (**a**), Phylogenetic analysis of *Le*Cho1. The neighbor-joining phylogenetic tree was constructed using MEGA 6.0 software. Chitosanases from the GH46, GH75, and GH80 families are separated by parentheses on the right. *Le*Cho1 is indicated in black bold font. (**b**), Sequence alignment of *Le*Cho1 with other chitosanases from the GH75 family. The dots indicate highly conserved amino acid residues typical of the GH75 family, among which blue dots indicate presumptive active site residues. Blue frames indicate conserved sites containing three or more identical amino acid residues in seven proteins. Black frames indicate distinct amino acid residues of *Le*Cho1 compared to other chitosanases from the GH75 family.

The putative chitosanase *Le*Cho1, encoded by an open reading frame consisting of 846 nucleotides, was predicted to be composed of 281 amino acid residues with a 22-aa signal peptide at its *N*-terminus. The mature chitosanase protein had a predicted molecular mass of 27.3-kDa and a calculated pI of 4.11. A phylogenetic tree including *Le*Cho1 and other chitosanases from the GH46, GH75, and GH80 families revealed that *Le*Cho1 clustered with the chitosanases from the GH75 family and formed an independent branch (Figure 1a). Multiple sequence alignment indicated that *Le*Cho1 exhibited low sequence identity with other members of the GH75 family. For example, it shared approximately 35% sequence identity with the chitosanases from *Penicillium chrysogenum* (ADG96019.1, 34.75%) and *Aspergillus oryzae* (BAD08218.2, 34.98%). Moreover, it only shared sequence identities of less than 25% with other chitosanases located in another branch of the GH75 family (Figure 1a). Nevertheless, it contained the four highly conserved amino acid residues (Asp140, Asp142, Asp204, and Glu215, indicated by dots in Figure 1b) typical of the GH75 family; among them, Asp204 and Glu215 (Figure 1b, indicated by blue dots) were predicted to be involved in the catalytic function of the enzyme. These results indicated that *Le*Cho1 was a novel member of the GH75 family.

### 3.2. Expression and Purification of LeCho1

**Figure 2:**
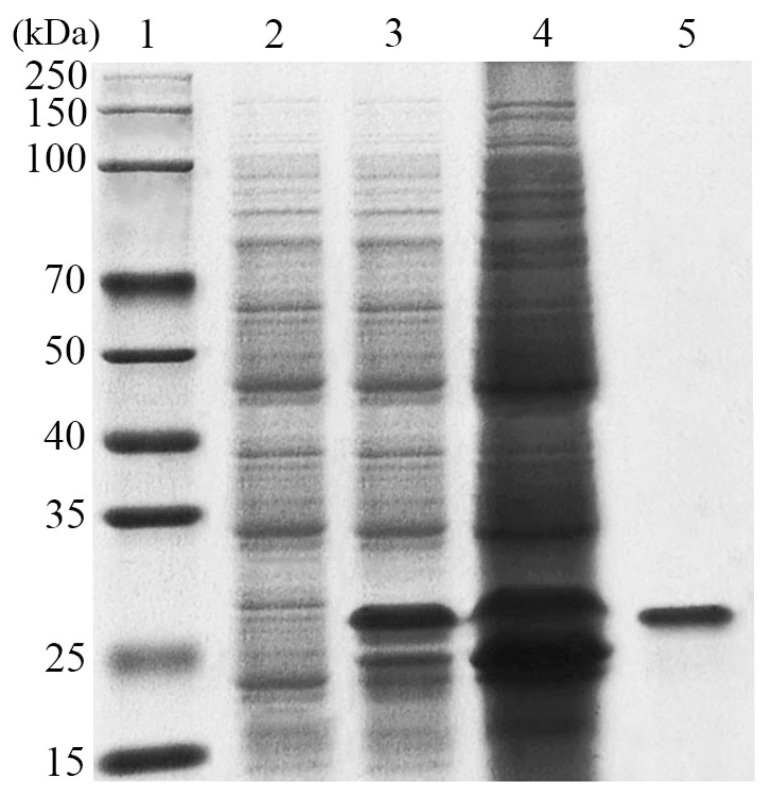
SDS-PAGE analysis of the purified recombinant *Le*Cho1 protein. Lane 1, standard molecular mass marker; lane 2, the supernatant of *E. coli* cells harboring pET29a (empty vector control) after ultrasonication; lane 3, the supernatant of *E. coli* cells harboring pET29a-*LeCHO1* (the recombinant plasmid) after ultrasonication; lane 4, the precipitate of *E. coli* cells harboring pET29a-*LeCHO1* after ultrasonication and centrifugation; lane 5, the purified *Le*Cho1 protein.

The coding sequence of the chitosanase without the signal peptide was successfully expressed in *E. coli* BL21 cells containing the recombinant plasmid pET29a-*LeCHO1*. The recombinant protein with a 6×His-tag, purified using a Ni-NTA affinity column, exhibited a single band on SDS-PAGE corresponding to a molecular weight of approximately 27-kDa (Figure 2), consistent with its calculated molecular mass of 27.3 kDa.

### 3.3. Substrate Specificity of the Purified Protein

The hydrolytic activity of *Le*Cho1 on different polysaccharides (chitosan, chitin, microcrystalline cellulose, and sodium carboxymethylcellulose) was examined in order to determine its substrate specificity. As shown in Table 1, *Le*Cho1 displayed high hydrolytic activity on chitosan, and its activity was gradually enhanced with the increase of the DDA, with the highest activity of 71.88 U/mg found for 95% deacetylated chitosan. However, it had no activity on chitin or cellulose, indicating the strict substrate specificity of *Le*Cho1 for chitosan.

### 3.4. Enzymological Characterization of LeCho1

**Figure 3:**
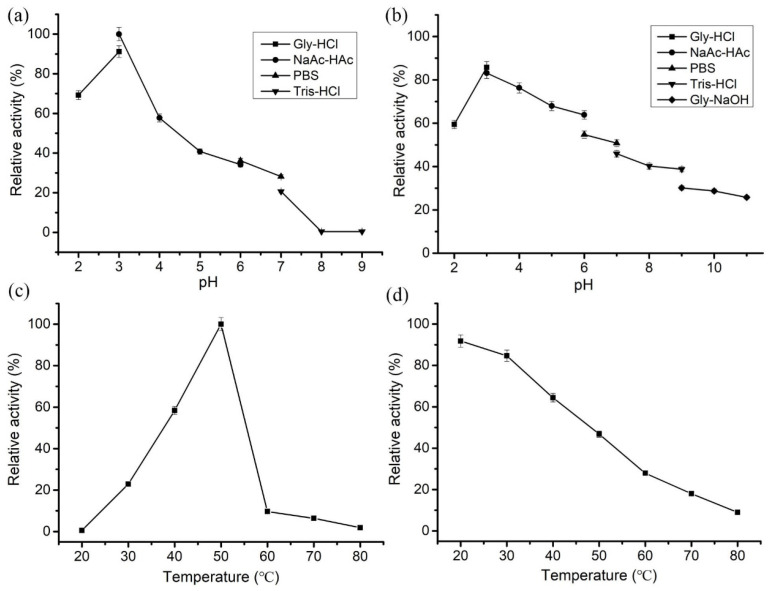
Biochemical characterization of purified *Le*Cho1. (**a**) Optimal pH of *Le*Cho1. (**b**) pH stability of *Le*Cho1. (**c**) Optimal temperature of *Le*Cho1. (**d**) Thermal stability of *Le*Cho1. The data represent the average of independent assays carried out in triplicate. The error bars indicate the standard deviations.

During the hydrolysis of chitosan, pH influences not only the activity and stability of the enzyme but also the conformation of the chitosan substrate itself. For example, chitosan has good solubility in an acidic environment while being insoluble under neutral or alkaline conditions. Thus, we investigated the effect of pH on the catalytic activity of *Le*Cho1 with chitosan (0.5% *w*/*v*, 95% DDA) as the substrate. *Le*Cho1 displayed the highest chitosanase activity in NaAc-HAc buffer pH 3.0 but also retained more than 40% relative activity at pH values ranging from 2 to 5. However, its activity decreased rapidly at pH above 6.0 with a complete loss of activity at pH 8.0 and 9.0 (Figure 3a), suggesting that *Le*Cho1 is an acid-adapted chitosanase. In addition, the enzyme still maintained more than 50% of the initial activity after incubation at pH 2.0–7.0 for 12 h. By contrast, it retained less than 40% of the initial activity after incubation in alkaline buffers (Figure 3b).

In addition, *Le*Cho1 showed maximal chitosanase activity at 50 °C, with more than 50% relative activity in the temperature range of 40–55 °C. However, its activity dropped sharply above 60 °C (Figure 3c). After incubation at 20–45 °C for 1 h, *Le*Cho1 retained more than 50% of the initial activity, compared to less than 30% at temperatures over 60 °C (Figure 3d), revealing that *Le*Cho1 was a mesophilic enzyme with favorable stability at temperatures below 45 °C.

In addition, the influences of different metal ions on the hydrolytic activity of *Le*Cho1 were also determined. As presented in Table 2, Mn^2+^ had an obvious stimulatory effect, increasing the enzyme activity by 57%. Additionally, Mg^2+^ and Ca^2+^ exhibited a slight promoting effect, with increases of approximately 15%. K^+^, Ba^2+,^ and Fe^2+^ had only a slight effect on the hydrolytic activity of *Le*Cho1. By contrast, Na^+^, Co^2+^, Ni^2+,^ and Zn^2+^ had a mild inhibitory effect on the activity of the enzyme. However, Fe^3+^ exhibited a strong inhibitory effect, resulting in the complete loss of chitosanase activity. As a metal chelating agent, EDTA had an inhibitory effect on the activity of *Le*Cho1, causing a 44% decrease at the concentration of 1 mM and 100% at 10 mM, indicating that *Le*Cho1 is a metalloenzyme.

**Figure 4:**
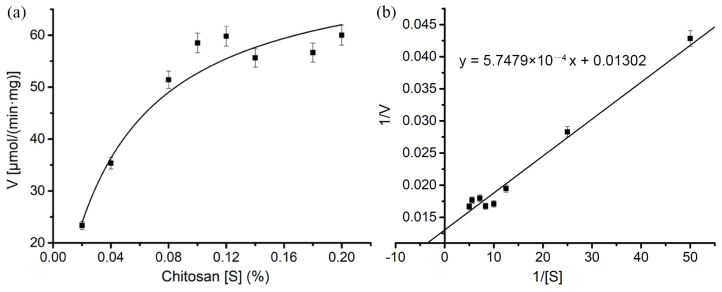
Enzyme kinetics of *Le*Cho1 were measured using different concentrations of 95% deacetylated chitosan in 50 mM Gly-HCl buffer (pH 3.0) at 40 °C. (**a**) Michaelis–Menten graph. (**b**) Lineweaver–Burk plot. The data represent the averages of independent experiments performed in triplicate. The error bars indicate the standard deviations.

To determine the kinetic parameters, the effect of the substrate concentration on the hydrolytic activity of *Le*Cho1 was determined using chitosan (95% DDA) in 50 mM Gly-HCl buffer (pH 3.0) at 40 °C. The results showed that the K_m_ and V_max_ values of *Le*Cho1 were 0.04 μM and 76.81 μmol·min^−1^·mg^−1^, respectively (Figure 4), suggesting a high affinity of the enzyme for the substrate.

### 3.5. LeCho1 Is an Endo-Chitosanase

**Figure 5:**
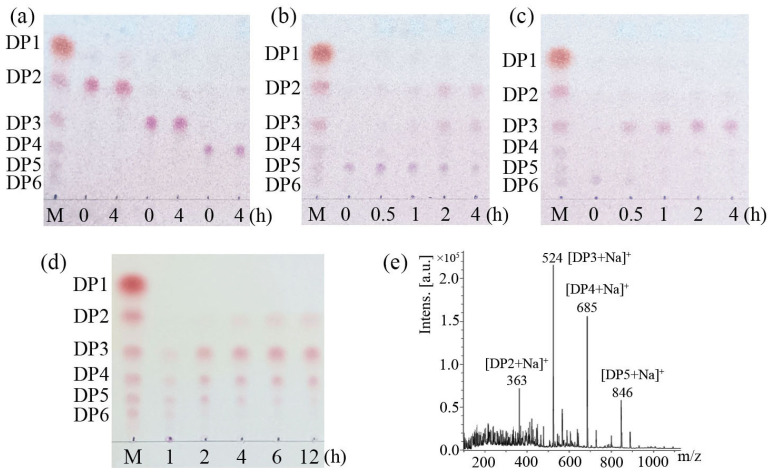
Analysis of the hydrolytic pattern of *Le*Cho1 with different substrates. M, standard chitooligosaccharides (GlcN, DP1–6). The substrates included chitobiose (**a**), chitotriose (**a**), chitotetraose (**a**), chitopentaose (**b**), chitohexaose (**c**), and 95% deacetylated chitosan (**d**). The hydrolysates were analyzed by TLC. (**e**) Mass spectrometry analysis of hydrolysates produced by *Le*Cho1 incubated with 95% deacetylated chitosan for 12 h.

The hydrolytic products of *Le*Cho1 released from chitooligosaccharides, including (GlcN)_2_, (GlcN)_3_, (GlcN)_4_, (GlcN)_5_, and (GlcN)_6_, as well as chitosan (0.5% *w*/*v*, 95% DDA), were detected using thin layer chromatography (TLC) and mass spectroscopy (MALDI-TOF MS). The TLC analysis showed no hydrolytic products when *Le*Cho1 was incubated with (GlcN)_2_, (GlcN)_3_, or (GlcN)_4_ (Figure 5a), suggesting that the enzyme had no activity on COSs with degrees of polymerization (DP) of less than 5. However, after incubation with *Le*Cho1 for 4 h, most of the (GlcN)_5_ was hydrolyzed into (GlcN)_2_ and (GlcN)_3_ (Figure 5b), while (GlcN)_6_ was completely degraded into (GlcN)_3_ (Figure 5c).

Additionally, *Le*Cho1 was also able to effectively hydrolyze chitosan. Time course analysis showed that hydrolysates of chitosan produced COSs with DP 3−6 after 1 h of reaction. After 2 h of reaction, there were large amounts of (GlcN)_3_, (GlcN)_4_ and (GlcN)_5_, with trace amounts of (GlcN)_6_ in the hydrolysate. As the reaction continued, (GlcN)_3_ and (GlcN)_4_ accumulated, while the content of (GlcN)_5_ and (GlcN)_6_ gradually decreased. After 12 h of reaction, the hydrolysates were mainly composed of (GlcN)_3_ and (GlcN)_4_, a small amount of (GlcN)_2_, and trace amounts of (GlcN)_5_ (Figure 5d). In addition, the hydrolysates produced by *Le*Cho1 incubated with chitosan for 12 h were analyzed using MALDI-TOF-MS. Fragments of ions of the products chitobiose ([DP2 + Na]^+^ at *m*/*z* 363), chitotriose ([DP3 + Na]^+^ at *m*/*z* 524), chitotetrose ( [DP4 + Na]^+^ at *m*/*z* 685), and chitopentaose ([DP5 + Na]^+^ at *m*/*z* 846) were present in the mass spectra (Figure 5e). These results indicated that *Le*Cho1 exhibited the endo-type catalytic mode of action in the hydrolysis of chitosan.

## 4. Discussion

The putative chitosanase *Le*Cho1 was cloned from *L. edodes* and identified as a novel member of the GH75 family. Phylogenetic analysis revealed that *Le*Cho1 clustered with other chitosanases from the GH75 family, and it also had the conserved catalytic amino acid residues Asp204 and Glu215 that are typical for the GH75 family [31]. However, *Le*Cho1 showed low sequence identity with other chitosanases belonging to the GH75 family, with identity values of less than 40%. In addition, we also found distinct amino acid residues near the active sites, such as Ala200 corresponding to a glycine (Gly) in other chitosanases (Figure 1b, indicated by the black frame) and Met202 corresponding to aromatic amino acids (Tyr, Phe and Trp) of other chitosanases (Figure 1b, indicated by the black frame), which were likely to contribute to its distinct enzymatic properties. To better assess the specific features of *Le*Cho1, we compiled the characteristics of previously reported chitosanases from the GH46, GH75, and GH80 families in Table 3. As can be seen, these enzymes possess molecular weights (Mw) in the range of 20.0–35.0 kDa, optimal temperatures between 40 and 50 °C, and optimal pH values ranging from 5.0 to 7.0 [32,33], as well as the ability to hydrolyze chitosan into COSs. Among them, *Le*Cho1 displayed some similarities with the chitosanases from the GH75 family in terms of molecular weight, optimal temperature, and hydrolytic profile [34,35,36]. However, *Le*Cho1 exhibited significant differences in terms of the optimal pH and influence of metal ions on its catalytic activity. Notably, the optimal pH of *Le*Cho1 was only 3.0, which was significantly more acidic than the optimal pH of other chitosanases. Additionally, the metal ion Mn^2+^ was found to enhance the hydrolytic activity of most chitosanases [23,37], whereby the activity of *Le*Cho1 was increased by 57%. By contrast, Cu^2+^ had an inhibitory effect on the activity of most chitosanases [18,38] but no effect on the activity of *Le*Cho1. In contrast, Fe^3+^ had an obvious inhibitory effect on the activity of *Le*Cho1, which was similar to reports on chitosanases from the GH46 and GH80 families, such as the enzymes from *Streptomyces coelicolor* A3(2) M145 [39] and *Bacillus* sp. BY01 [18]. 

As a promising novel enzyme, *Le*Cho1 has potential applications in the production of bioactive chitooligosaccharides. In contrast to their precursor, chitosan, COSs possess low molecular weight and high solubility in water. They are widely used in many domains, especially in agricultural cereal crop production, which provides the basic guarantee for improving the quality of food raw materials. COSs with desirable DP were reported to act as biostimulants that regulate plant growth and development. Zhang et al. (2016) [40] found that COSs with DP > 3 induced an increase in the contents of soluble protein, soluble sugar, and chlorophyll in wheat seedlings, promoting photosynthesis and plant growth. Moreover, COSs could enhance plant resistance to abiotic stresses such as salinity, drought, heavy metals, and heat due to their ability to scavenge reactive oxygen species. Jia et al. (2019) [41] reported that chitotriose and chitotetraose promoted the health of soybean seedlings at 72 and 42 h of germination, which was attributed to their radical scavenging ability. In addition, COSs were also investigated as a green biocide against phytopathogenic fungi due to the binding of their positively charged amino groups (NH_3_^+^) to negatively charged constituents of the fungal cell wall [42], which causes clustering and lysis of fungal mycelia. Wang et al. (2021) [15] discovered that COSs with DP 3–5 were able to inhibit the mycelial growth of *Fusarium oxysporum* and *Magnaporthe oryzae* at high concentrations. In this study, *Le*Cho1 was found to effectively cleave chitosan, exhibiting a specific activity of 71.88 U/mg. Moreover, the hydrolysate obtained after 2 h was primarily composed of COSs with DP 3–5 and contained no COSs with a DP of less than 3, giving *Le*Cho1 high competitiveness in the manufacture of desirable COSs. Furthermore, *Le*Cho1 exhibited maximal catalytic activity under acidic conditions (pH 3.0), which is also a major advantage in the preparation of COSs, as low pH is helpful not only in reducing the risk of microbial contamination but also in enhancing the solubility of chitosan and further improving hydrolysis efficiency. Moreover, *Le*Cho1 exhibited a low Michaelis constant, with a *K*_m_ value of 0.04 μM, indicating that the enzyme has a high affinity for chitosan, which is also beneficial for the preparation of COSs. In addition, the expression of the chitosanase *Le*Cho1 was based on the well-known *E. coli* BL21 (DE3) expression system, with the advantages of a short fermentation period, high production efficiency, and mature purification process. In short, the efficient expression of *Le*Cho1 and its outstanding enzymatic properties render it a good candidate for the industrial production of chitooligosaccharides.

## 5. Conclusions

In this study, the chitosanase *Le*Cho1 from *L. edodes* was identified through bioinformatics analysis, expressed in *E. coli*, purified, and characterized. *Le*Cho1 was revealed to be a new member of the GH75 family. The purified recombinant enzyme was most effective on 95% deacetylated chitosan, with a specific activity of 71.88 U/mg. *Le*Cho1 was most active at pH 3.0 and 50 °C, with good stability at pH values ranging from 2.0 to 6.0 and temperatures below 40 °C. Its *K*_m_ and *V*_max_ values were 0.04 μM and 76.81 μmol·min^−1^·mg^−1^, respectively, indicating high affinity for the substrate chitosan. *Le*Cho1 exhibited endo-type chitosanase activity, catalyzing the depolymerization of chitosan to yield COSs with DP 2–5. The remarkable properties of *Le*Cho1 indicate its promising application in the production of bioactive COSs.

## Figures and Tables

**Table 1:** Substrate specificity of the purified *Le*Cho1 protein.

Substrate	Specific Activity (U/mg)
Chitosan (DDA 70%)	37.18 ± 1.28 ^c^
Chitosan (DDA 80%)	50.22 ± 1.73 ^b^
Chitosan (DDA 95%)	71.88 ± 2.48 ^a^
Chitin	0 ^d^
Microcrystalline cellulose	0 ^d^
Sodium carboxymethylcellulose	0 ^d^

The data are expressed as the mean ± standard error. The values with different letters were significantly different at *p* < 0.05, according to Duncan’s test.

**Table 2:** Effects of metal cations and chelating agent EDTA on *Le*Cho1 activity.

Metal Ions	Concentration (mM)	Relativity Activity (%)
No addition	0	100 ± 2.11 ^c^
Na^+^ (NaCl)	1	95.93 ± 3.09 ^d^
K^+^ (KCl)	1	107.89 ± 3.48 ^c^
Ba^2+^ (BaCl_2_)	1	108.46 ± 3.50 ^c^
Cu^2+^ (CuCl_2_)	1	98.94 ± 2.72 ^c^
Ca^2+^ (CaCl_2_)	1	110.25 ± 3.56 ^b^
Co^2+^ (CoCl_2_)	1	90.32 ± 2.91 ^e^
Fe^2+^ (FeCl_2_)	1	107.32 ± 3.46 ^c^
Mn^2+^ (MnCl_2_)	1	156.63 ± 5.05 ^a^
Mg^2+^ (MgCl_2_)	1	118.71 ± 3.83 ^b^
Ni^2+^ (NiCl_2_)	1	94.87 ± 3.06 ^d^
Zn^2+^ (ZnCl_2_)	1	95.69 ± 3.09 ^d^
Fe^3+^ (FeCl_3_)	1	0.00 ± 0.00 ^h^
Al^3+^ (AlCl_3_)	1	85.19 ± 2.75 ^f^
Cd^3+^ (CdCl_3_)	1	83.32 ± 2.69 ^f^
EDTA	1	56.17 ± 1.78 ^g^
	10	0.00 ± 0.00 ^h^

The data are shown as the mean ± standard error. The values with different letters were significantly different at *p* < 0.05, according to Duncan’s test.

**Table 3:** Characteristics of the chitosanase from different sources.

Microorganism	Chitosanase	Family	Mw (kDa)	Optimal Temperature (°C)	Optimal pH	Metal Ions (+)	Metal Ions (−)	End Products	Reference or Source
*Lentinula edodes*	*Le*Cho1	GH75	27	50	3.0	Mn^2+^	Fe^3+^	DP2–5	This study
*Aspergillus* sp. W-2	CsnW2	GH75	28	55	6.0	Mn^2+^, Ca^2+^, Mg^2+^	Cu^2+^, Fe^2+^, Zn^2+^, Ni^2+^, Ge^2+^	DP2–6	[34]
*Aspergillus fumigatus* CJ22-326	Csn75	GH75	23.5	55–65	5.0–6.0	Mn^2+^	Cu^2+^, Mg^2+^	DP2–4	[35]
*Beauveria bassiana*	BbCSN-1	GH75	33	30	5.0	Mn^2+^	Cu^2+^, Co^2+^	DP2–3	[36]
*Streptomyces coelicolor* A3(2) M145	CsnA	GH46	26	65	6.0	Mn^2+^, Co^2+^	Fe^3+^, Al^3+^, Ni^2+^	DP2–3	[39]
*Paenibacillus elgii* TKU051	chitosanase	GH46	28	55	6.0	Mn^2+^	Cu^2+^	DP2–3	[23]
*Streptomyces hygroscopicus* R1	ShCsn46	GH46	28	55	5.5	Mn^2+^	Cu^2+^, Fe^2+^, Al^3+^	DP2–6	[37]
*Bacillus* sp. BY01	CsnB	GH46	30	35	5.0	Mn^2+^, Cu^2+^, Mg^2+^	Fe^3+^	DP2–3	[18]
*Matsuebacter chitosanotabidus* 3001	ChoA	GH80	34	30–40	4.0	ND	Ag^+^	DP2–6	[32]
β-*Proteobacterium* KNU3	ChoK	GH80	34	70	6.0	Cu^2+^, Co^2+^	Ca^2+^, Zn^2+^, Mg^2+^	ND	[33]
Mitsuaria sp. 141	ChoA	GH80	33	65	5.5	Fe^2+^	Cu^2+^, Hg^2+^	DP1–5	[38]

Mw: molecular weight. Metal ions (+): the metal ions promoting the activity of chitosanase. Metal ions (−): the metal ions inhibiting the activity of chitosanase. ND: not detected.

## Data Availability

The original contributions presented in the study are included in the article, further inquiries can be directed to the corresponding author.
